# Do Vibram Fivefingers® really mimic barefoot conditions? A study examining walking efficiency

**DOI:** 10.1186/1757-1146-3-S1-O9

**Published:** 2010-12-20

**Authors:** Sarah Curran, Joanna Tozer

**Affiliations:** 1 University of Wales Institute, Cardiff, UK

## 

Although the role of Vibram Fivefingers^®^ (minimal footwear) in running is gaining in popularity, the influence of this style of footwear on walking and energy efficiency is currently unknown. The purpose of this study was to determine if energy efficiency was similar in barefoot walking compared to wearing Vibram Fivefingers^®^. Fifteen participants (8 females, 7 males, age range 21 – 48 years) walked on a treadmill for 20 minutes at a speed of 4.2km/hour (0% incline) in two randomly assigned conditions: barefoot and Vibram Fivefingers^®^. Heart rate, (HR), oxygen consumed in litres per kilogram (VO^2^/kg), respiration exchange ratio (RER), number of steps (NoS) and the physiological cost index (PCI) were measured for each condition. The Footwear Comfort Scale was completed after wearing Vibram Fivefingers^®^. There were no significant differences (p>0.05) between HR, VO^2^/kg, RER, NoS and PCI between each condition. The areas of medio-lateral control, heel cup fit and shoe length were identified by the Footwear Comfort Scale as the least comfortable. The findings of this study suggest that Vibram Fivefingers^®^ are equal in terms of energy efficiency when compared to barefoot walking. Further research is required to explore the role of minimal footwear during other weightbearing activities.

